# Raman spectroscopy of a near infrared absorbing proteorhodopsin: Similarities to the bacteriorhodopsin O photointermediate

**DOI:** 10.1371/journal.pone.0209506

**Published:** 2018-12-26

**Authors:** Gaoxiang Mei, Natalia Mamaeva, Srividya Ganapathy, Peng Wang, Willem J. DeGrip, Kenneth J. Rothschild

**Affiliations:** 1 Molecular Biophysics Laboratory, Photonics Center and Department of Physics, Boston University, Boston, Massachusetts, United States of America; 2 Department of Biophysical Organic Chemistry, Leiden Institute of Chemistry, Leiden UniversityAR Leiden, The Netherlands; 3 Bruker Corporation, Billerica, MA, United States of America; Boston Medical Center, Boston University School of Medicine, UNITED STATES

## Abstract

Microbial rhodopsins have become an important tool in the field of optogenetics. However, effective *in vivo* optogenetics is in many cases severely limited due to the strong absorption and scattering of visible light by biological tissues. Recently, a combination of opsin site-directed mutagenesis and analog retinal substitution has produced variants of proteorhodopsin which absorb maximally in the near-infrared (NIR). In this study, UV-Visible-NIR absorption and resonance Raman spectroscopy were used to study the double mutant, D212N/F234S, of green absorbing proteorhodopsin (GPR) regenerated with MMAR, a retinal analog containing a methylamino modified β-ionone ring. Four distinct subcomponent absorption bands with peak maxima near 560, 620, 710 and 780 nm are detected with the NIR bands dominant at pH <7.3, and the visible bands dominant at pH 9.5. FT-Raman using 1064-nm excitation reveal two strong ethylenic bands at 1482 and 1498 cm^-1^ corresponding to the NIR subcomponent absorption bands based on an extended linear correlation between λ_max_ and γ_C = C_. This spectrum exhibits two intense bands in the fingerprint and HOOP mode regions that are highly characteristic of the O_640_ photointermediate from the light-adapted bacteriorhodopsin photocycle. In contrast, 532-nm excitation enhances the 560-nm component, which exhibits bands very similar to light-adapted bacteriorhodopsin and/or the acid-purple form of bacteriorhodopsin. Native GPR and its mutant D97N when regenerated with MMAR also exhibit similar absorption and Raman bands but with weaker contributions from the NIR absorbing components. Based on these results it is proposed that the NIR absorption in GPR-D212N/F234S with MMAR arises from an O-like chromophore, where the Schiff base counterion D97 is protonated and the MMAR adopts an all-*trans* configuration with a non-planar geometry due to twists in the conjugated polyene segment. This configuration is characterized by extensive charge delocalization, most likely involving nitrogens atoms in the MMAR chromophore.

## Introduction

Microbial rhodopsins (classified as Type 1 rhodopsins) are retinal containing, seven-helix transmembrane proteins that absorb UV and visible light. One of the best known examples is bacteriorhodopsin (BR), which has a visible λ_max_ near 570 nm and functions as a light-driven proton pump [1, 2]. Using a combination of biophysical techniques including static and laser-flash transient visible absorption spectroscopy, FTIR-difference and resonance Raman spectroscopy (RRS), solid-state NMR, cryo-electron microscopy and x-ray crystallography, a relatively detailed picture of the BR proton pumping mechanism has emerged [3–9] including the mechanism of color tuning [10–12].

**Fig 1:**
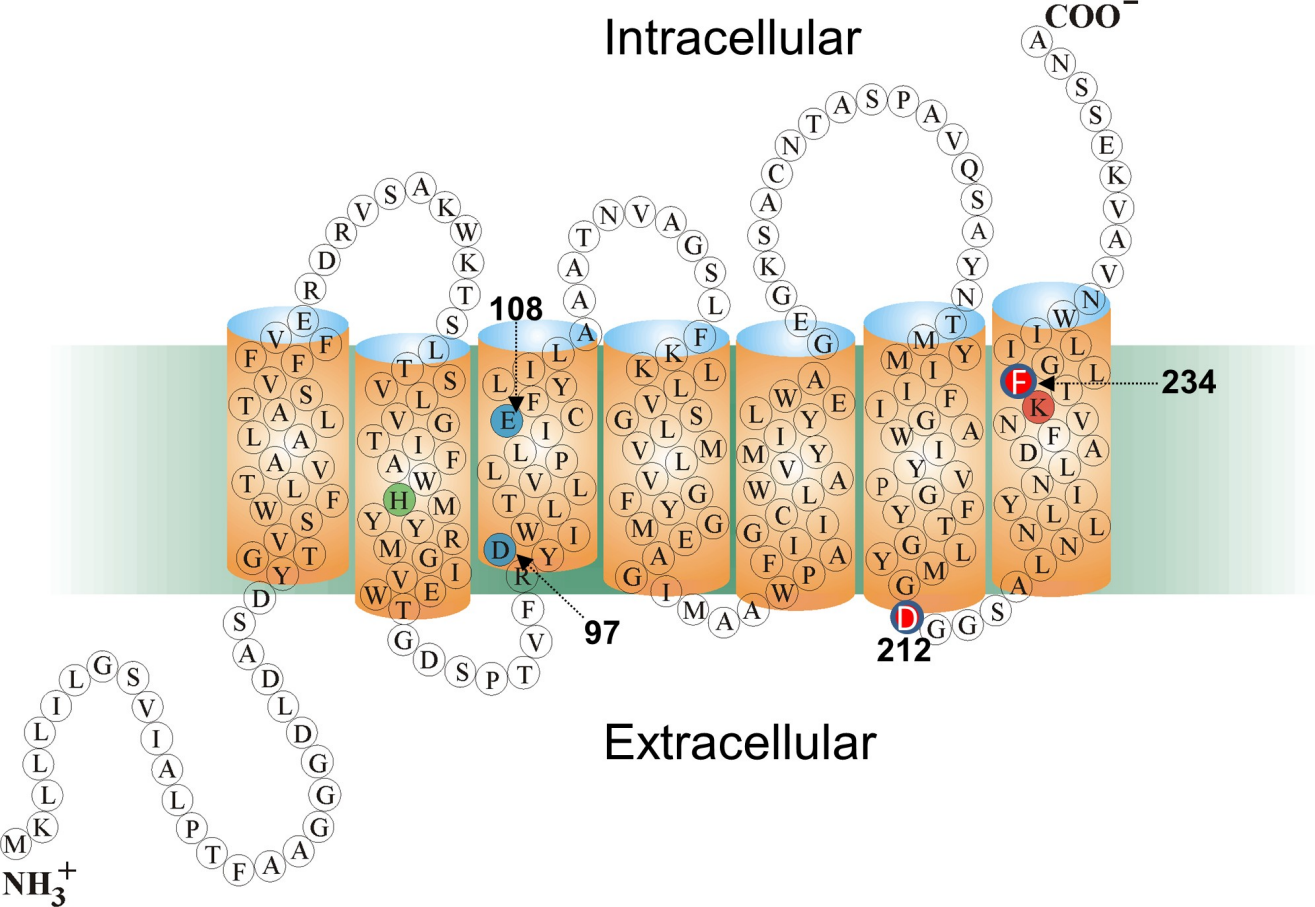
Predicted 2D folding pattern of GPR in bilayer membrane along with key residues in sequence. Sequence numbering given is for the Monterey Bay eBAC31A08 variant of GPR. The key carboxylate groups Asp97 and Glu108 (blue), Schiff base-forming residue Lys231 (orange) and His75 (green) are highlighted. The two residues Asp212 and Phe234 which are substituted with an Asn and Ser, respectively, to form GPR-DNFS are shown in red with white lettering. Reprinted in modified form from [22] under a CC BY license, with permission from the American Society for Biochemistry and Molecular Biology.

In addition to archaea, microbial rhodopsins have been found in bacteria and eukarya, the other two major domains of life, [13–15]. For example, proteorhodopsins (PRs) which have diverse functions including serving as light-driven proton pumps, were discovered in marine proteobacteria and are ubiquitous throughout the world’s oceans [16–19]. The visible absorption maxima of PRs are clustered near 520 nm (green proteorhodopsin; GPR) or 490 nm (blue proteorhodopsin; BPR) [18, 20, 21]. They all share with BR several key conserved amino acid residues including Asp97 in helix C (Asp85 in BR), which functions as the Schiff base (SB) counterion and proton acceptor, Glu108 (Asp 96 in BR), the Schiff Base (SB) proton donor and Lys231 in helix G (Lys216 in BR) which forms a SB with the retinylidene chromophore (Fig 1). Other residues such as His75 (helix B) have no counterpart in BR and may serve as part of a proton relay mechanism through direct interaction with Asp97 [22–24], as well as in inter-subunit interaction in oligomer formation [23].

Besides intrinsic interest in elucidating molecular differences between the BR and PR proton pump mechanisms, PRs have been engineered for use in a variety of biotechnological applications. For example, a bioengineered PR has been used in *E*. *coli* to produce a proton based electrochemical gradient to power chemotaxis and generate byproducts such as biohydrogen [25–27]. Introduction of the mutation D97N in GPR (GPR-D97N:A1) transforms it from a proton pump to a fluorescent nanosensor of transmembrane voltage [28]. When expressed in *E*. *coli*, this mutant led to the discovery of electrical spiking which has subsequently been associated with calcium influx [29, 30]. Similar visible absorbing fluorescent voltage nanosensors have been extensively bioengineered for mammalian cellular expression including QuasARs [31], Archers [32] and more recently Archons [33].

Despite these advances, effective *in vivo* optogenetic monitoring and control of neural activity using microbial rhodopsins is in large part severely limited because: 1) biological tissues strongly absorb and scatter visible light, and 2) until recently the absorbance bands of all known microbial rhodopsins and mutants did not extend into the NIR region (see below). Thus, *in vivo* real-time imaging, ideally at the single neuron level, of the electrical activity of complex circuits located below the surface layers of the brain are exceptionally difficult to perform without the use of implantable optical fibers or electrodes. In fact, *in vivo* deep brain imaging of neural activity is especially important to study the basis of neurodegenerative and neuropsychiatric disorders [34].

Recently, progress has been made in this direction by shifting the absorption of a GPR into the near infrared (NIR), while maintaining proton pump activity [35]. This was accomplished by: i) Utilizing the red-shifted double mutant D212N/F234S (GPR-DNFS) (Fig 1) which was discovered by screening random PCR mutants of GPR and was identified as the most red-shifted mutant retaining activity (shift from 548 nm to 562 nm of the protonated form in detergent micelles [36]; note almost all of this red-shift is due to the F234S substitution [36]); ii) Substitution of the native A1 retinal with the analog retinal 3-methylamino-16-nor-1,2,3,4-didehydroretinal (MMAR) to form GPR-DNFS:MR (S1 Fig). This combination resulted in a dramatic red-shift of approximately 200 nm from the native visible absorption of GPR with A1 retinal (GPR:A1) into the NIR [35]. The resulting broad absorbance band has a strong component around 740 nm, which is further enhanced upon protonation of the counterion Asp97 [35]. However, a detailed understanding of the chromophore structure of GPR-DNFS:MR and the molecular basis for the large red-shift is still lacking. Recently, a similar approach has also been reported using mutants of archaerhodopsin-3 (AR3) regenerated with merocyanine retinal analogs [37]. This resulted in strongly fluorescent AR3 variants (peak emission around 710 nm), which, however, lost their proton pump capacity [37].

Resonance Raman spectroscopy provides an effective means to probe retinal chromophores covalently attached to microbial and animal rhodopsins. The wavelength shift induced by the inelastic scattering of photons from various vibrational modes of the chromophore, an effect which is resonance enhanced when the exciting wavelength overlaps with the absorption band(s) of the chromophore, provides valuable information about the local environment, ionization state and configuration of the retinal [38–42]. However, since visible light can activate the photocycle of microbial rhodopsins such as GPR due to spectral overlap with the visible absorption band, various techniques including the use of flow and spinning cells have been developed to selectively probe the unphotolyzed state or a particular photocycle intermediate [43–45]. The use of NIR excitation wavelengths such as 1064-nm in combination with FT-Raman largely avoids this problem while still producing significant enhancement of the retinal vibrational bands due to pre-resonance conditions [46, 47]. Additional techniques such as stimulated Raman scattering [48] can also be used with NIR Stokes and pump wavelengths to probe the vibrational spectrum of the chromophore of microbial rhodopsin as demonstrated recently for GPR:MR [49].

In this study, we explore the properties of GPR-DNFS:MR and related PRs regenerated with MMAR using a combination of UV-Vis-NIR absorption and RRS. Four distinct subcomponent absorption bands are found in GPR-DNFS:MR. Two bands in the visible (~560 and ~620 nm) dominate at high pH (≥ 9.5), while two bands in the NIR (~710 and ~780 nm) dominate at lower pH (≤ 8). Similar absorption subcomponent bands were also found to exist in the absorption of GPR:A1 and its D97N mutant regenerated with MMAR (GPR-D97N:MR). Raman spectroscopy reveals that the NIR species exhibit spectral features, which are very similar to that of the O photointermediate of the light-adapted BR (BR_570_) photocycle, while a ~560 nm absorbing species shows similarities to BR_570_ as well as acid-purple membrane. By analogy with BR, all spectral components of GPR-DNFS:MR appear to possess an all-*trans* configuration of the chromophore with a PSB, but the NIR species also have a distorted non-planar polyene structure.

## Materials and methods

### Expression, purification and reconstitution of GPR-DNFS:MR, GPR:MR and GPR-D97N:MR into membrane vesicles

Methods for the expression, purification and reconstitution of GPR-DNFS:MR, GPR:MR and GPR-D97N:MR in model lipid membrane vesicles were similar to methods previously reported for GPR and AR3 [35, 47]. BL21(DE3) *E*. *coli* competent cells were used for expression of these proteins (Millipore, Billerica, MA, Cat #69450–3). Plasmids encoding GPR (wild type, WT), D212N/F234S with a C-terminal His tag were produced as previously described [35]. The plasmid for D97N (PROPS) was a generous gift from J. Kralj which also contained a C-terminal His-tag. All-*trans* retinal was purchased from TRC company, Canada and all-*trans*-3-methylamino-16-nor-1,2,3,4-didehydroretinal (MMAR; purity > 99.9%) was custom synthesized by Buchem, B.V. Retinals were stored at -80°C in an ethanol stock solution. Octylglucoside (OG) and n-dodecyl-β-D-maltoside (DDM) were purchased from Anatrace Products, OH. Briefly, *E*. *coli (*strain BL21 (DE3), pet28b(+) plasmid with the WT or the D212N/F234S gene were grown in 0.5 L of LB medium with 50 mg/L ampicillin, to an O.D of 0.4 at 600 nm at 32°C. All-*trans* retinal (2 μM) or all-*trans-*MMAR (1 μM) and inducer (IPTG, 1 mM) were added and cells were grown for an additional 20 h in the dark at 32°C. Cells were then harvested by centrifugation using a Beckman-Coulter Spinchron DLX tabletop centrifuge at 3,000 RPM (~860 g), resuspended in sonication buffer (50 mM Tris, 5 mM MgCl_2_ at pH 7.0), and lysed by freeze-thaw followed by sonication of the sample on ice for 1 minute, 3 times. The lysate was then centrifuged at 38,000 RPM (~63,800g) with a Beckman-Coulter Optima L-90K ultracentrifuge with a 70 Ti rotor, and the pellet resuspended in binding buffer (20 mM HEPES, 150 mM NaCl,10 mM imidazole; pH 7.0). The mixture was homogenized with a glass Wheaton homogenizer, 1.5% OG or 2% DDM added, and incubated at 4°C overnight using a rotatory shaker, and again centrifuged for 30 min. at 27,000 RPM (~32,000g) using the Beckman-Coulter Optima L-90K ultracentrifuge. Ni-NTA Agarose (QIAGEN) beads were washed with the binding buffer, added to the supernatant and incubated 2 h at 4°C using a rotary shaker. Nickel chelated nitrotriacetic acid (Ni-NTA) agarose beads with bound protein were loaded into 3 mL disposable plastic column and washed with 5 mL of wash buffer (50mM HEPES, 100 mM NaCl, 10 mM imidazole, 1% OG; pH 7.0). Protein was eluted with 1.5 mL of elution buffer (50 mM HEPES, 100 mM NaCl, 1% OG, 400 mM imidazole; pH 7.0). Purified His-tagged GPR or variants was reconstituted in *E*. *coli* polar lipids (ECPL) (Avanti, Alabaster AL) at 1:10 protein-to-lipid (w/w) ratio. Lipids were dissolved at 5mg/mL by sonication in binding buffer with 1% OG followed by filtration. The lipid solution was incubated with the OG solubilized protein for 15min at 4°C and dialyzed against the dialysis buffer (50 mM K_2_HPO_4_, 300 mM NaCl pH 7.0) overnight at 4°C followed by a buffer change and an additional dialysis for 3 h. The reconstituted protein was centrifuged for 3 min at 15K rpm and resuspended in 5 mM K_2_HPO_4,_ 100 mM NaCl, pH 7.0 buffer 3 times. GPR and variants samples were stored at 4°C. All procedures were the same for pBAD D97N (PROPS) plasmid except 1% L-arabinose was substituted for IPTG as an inducer.

### UV-Vis-NIR absorption spectroscopy

The protein samples for absorption measurements were prepared as previously reported [50–52] using approximately 50 μg of the protein in the form of reconstituted ECPL lipid membranes as described above. The samples were washed at least three times in approximately 0.1 mL of buffer (pH 5 buffer: 5mM NaH_2_PO_4_,10mM NaCl, 10mM MES; pH 7.3 buffer: 50mM NaCl, 5mM HEPES; pH9.5 buffer: 50mM NaCl, 10mM CHES). After the final wash, the supernatant was removed, and the sample resuspended in 50 μL of the above described buffer. The samples were then deposited on BaF_2_ windows and slowly dried in a dry-box for approximately 30 min. Samples were then rehydrated through the vapor phase with a small drop (~ 0.5 μL) of H_2_O and sealed in a sample cell with another BaF_2_ window. UV-Vis-NIR absorption measurements were performed at room temperature on a Cary 50 instrument after leaving the sample in the dark for 30 min. The samples were scanned at a rate of 600 nm/min over the range 200–1100 nm.

### Raman spectroscopy

Reconstituted GPR:MR, GPR-DNFS:MR and GPR-D97N:MR membrane vesicles and variants were prepared for Raman spectroscopy as described previously [53]. Approximately 30 μg of the reconstituted sample was spun in a SCILOGEX D3024 centrifuge at 15,000 rpm for 5 min, and the resulting pellet was re-suspended in the same wash buffer as described above for the different pH values. The solution was then re-pelleted and washed at least 2 additional times to form a final pellet. The final pellet was resuspended in a small amount of the wash buffer (< 5 μL) and transferred using a 10 μL syringe (Hamilton Company, Reno, NV) to a 0.5mm ID square borosilicate glass capillary (Fiber Optic Center, New Bedford, MA) with one end sealed. The capillary was spun at a lower speed (10,000 RPM, 3 min), then the open side was sealed with Critoseal (Leica Microsystems, Buffalo Grove, IL).

FT-Raman measurements using 1064-nm excitation were obtained on a Bruker MultiRam FT-Raman spectrometer equipped with a Ge detector operating at 4 cm^-1^ resolution and power ranging from 100–300 mW. RRS measurements using 532-nm excitation were obtained at room temperature on a Renishaw inVia confocal Raman microscope equipped with a CCD detector, a 20x objective with numerical aperture (NA) of 0.4. and power of approximately 2.8 mW and effective pixel resolution of ~1.2 cm^-1^. The system calibrates frequency accuracy using the 520.9 cm^-1^ band from an internal silicon chip. In addition, calibration was performed by recording the Raman spectrum of a control acetaminophen sample. Data acquisition consisted of a series of measurement cycles with each cycle consisting of 1 second data acquisition period followed by a 5 second wait-time in the dark. Depending on the signal-to-noise ratio, this cycle was repeated 100 to 1000 times. The spectra of the data acquisition period were then averaged. The empty capillary spectrum was subtracted from the averaged spectra to remove the fluorescence background. A multi-point linear baseline correction was performed to obtain the final reported spectra.

### Spectral analysis

Spectral subtractions, baseline corrections, Fourier self-deconvolution and peak fitting were all performed using GRAMS/AI v7.02 (Thermo Fisher Scientific, Inc.). This software package, which incorporates iterative chi-squared minimization, was also used to fit the subcomponent bands in the ethylenic and SB region of the FT-Raman and in the visible absorption spectrum. FT-Raman spectra were fitted from 1420–1660 cm^-1^ and 1100–1280 cm^-1^ with a linear baseline. Initial peak positions were determined using Fourier self-deconvolution. For the 1420–1660 cm^-1^ and the 1100–1280 cm^-1^ regions, the curve fitting procedure found 9 and 10 Voigtian peaks, respectively, which resulted in R^2^ values better than 0.99. The same program was used for curve fitting the UV-Vis spectrum in the range 475–850 nm resulting in 4 Voigtian peaks with R^2^ value better than 0.99.

## Results

### UV-Vis-NIR Absorption of GPR-DNFS:MR

**Fig 2:**
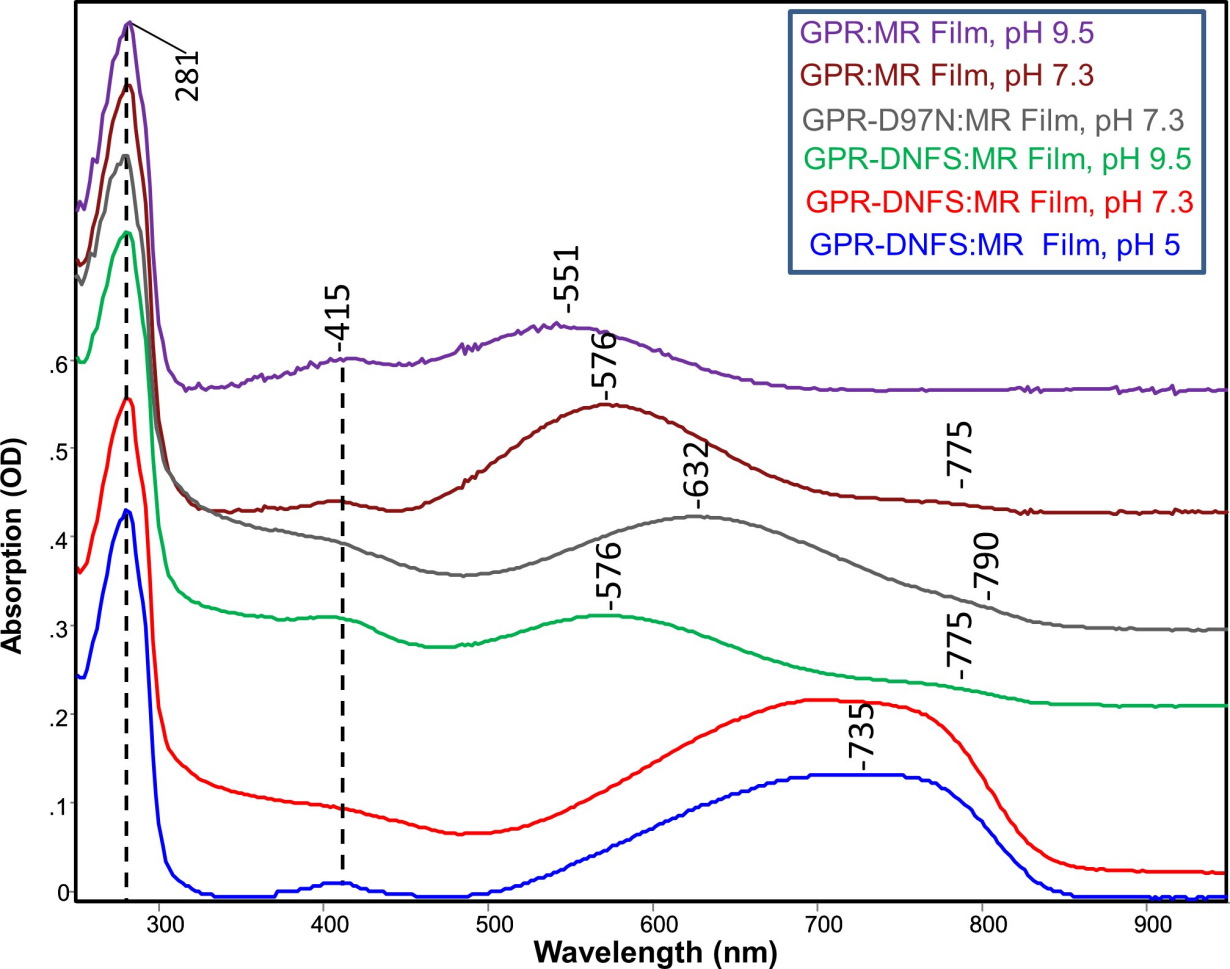
Absorption spectra from 250–950 nm of GPR and mutants containing MMAR chromophore at different pHs. Spectra were recorded at room temperature of GPRs reconstituted into *E*. *coli* polar lipids membrane vesicles and used to produce fully hydrated multilamellar films deposited on BaF_2_. All spectra were scaled using the 281 nm absorption band. DNFS is abbreviation for the mutant D212N/F234S and MR for MMAR chromophore. Absorption (OD) scale shown is for the GPR-DNFS:MR film at pH 5.

The absorption spectrum in the 250–950 nm region of the mutant GPR-D212N/F234S regenerated with MMAR (GPR-DNFS:MR) reconstituted into ECPL lipid membrane at pH 5, 7 and 9.5 and deposited as a hydrated multilamellar film onto a BaF_2_ window which is part of a sealed cell is shown in Fig 2 (see Materials and Methods). All spectra are normalized using the 281 nm band originating from the UV absorption of aromatic residues (Phe, Tyr and Trp) [54]. In agreement with previous absorption measurements of GPR-DNFS:MR in detergent micelles (n-dodecyl-β-D-maltoside (DDM)) [35], the major absorption band is significantly broadened at pH 5 and 7.3 and red-shifted over 200 nm into the NIR region near 735 nm when compared to native GPR with A1 retinal (GPR:A1) with absorbance maxima of 525 nm at pH 7.3 (S2 Fig) and 548 nm at pH 5 [25, 36]. It can also be surmised that the absorption in the NIR region of GPR-DNFS:MR consists of at least two components based on the broadness and asymmetry of the peak. In contrast, at pH 9.5 GPR-DNFS:MR exhibits a major band near 576 nm, only about 50 nm red-shifted from GPR:A1 at alkaline pH [17, 18, 20, 25, 36] along with a very weak band near 775 nm (Fig 2). A weak band near 415 nm also appears for all GPRs regenerated with MMAR which is likely to arise from residual cytochrome impurities that are often difficult to remove during *E*. *coli* membrane protein purification using Ni-NTA agarose His-tag affinity chromatography [25, 55].

**Fig 3:**
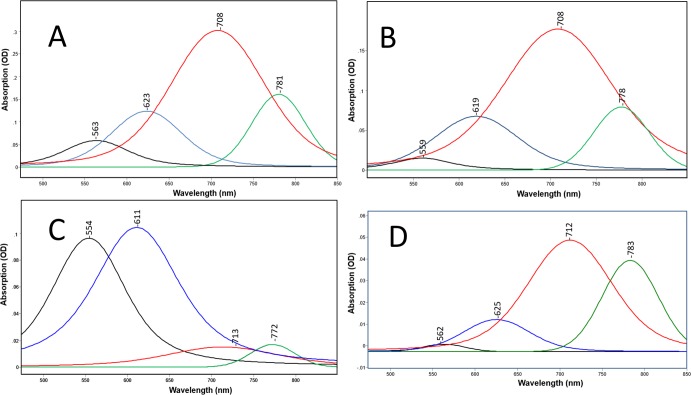
A-D. Curve fitted components of absorption spectra shown in Fig 2 for GPR-DNFS:MR at different pHs. (A) GPR-DNFS:MR at pH 5 in hydrated film; (B) GPR-DNFS:MR at pH 7.3 in hydrated film; (C) GPR-DNFS:MR at pH 9.5 in hydrated film; and (D) same as B but suspension measured in aqueous buffer (see Materials and Methods). All spectra are scaled to the largest component peak. Curve fitted components are colored black, blue, red and green from lowest to highest wavelength of component peak maxima. See Materials and Methods for details of curve fitting method.

Curve fitting (see Materials and Methods) reveals that there are at least 4 subcomponent bands that comprise the absorption band of GPR-DNFS:MR (Fig 3A–3C). At pH 5 and 7 bands appear near 560, 620, 710 and 780 nm (Fig 3A and 3B). The ~710 nm band is the most intense by at least a factor of 2 with a band-width of 109 nm. In comparison, at pH 9.5, bands still appear at similar wavelengths (Fig 3C) but the visible bands are far more intense. GPR-DNFS:MR membrane vesicles measured in aqueous buffer at pH 7.3 (see Materials and Methods) exhibit a similar band composition as the film spectrum, although the component near 780 nm increases in intensity relative to the band near 710 nm as well as to the visible bands (Fig 3D vs 3B) (see Discussion).

### Raman spectroscopy of GPR-DNFS:MR

**Fig 4:**
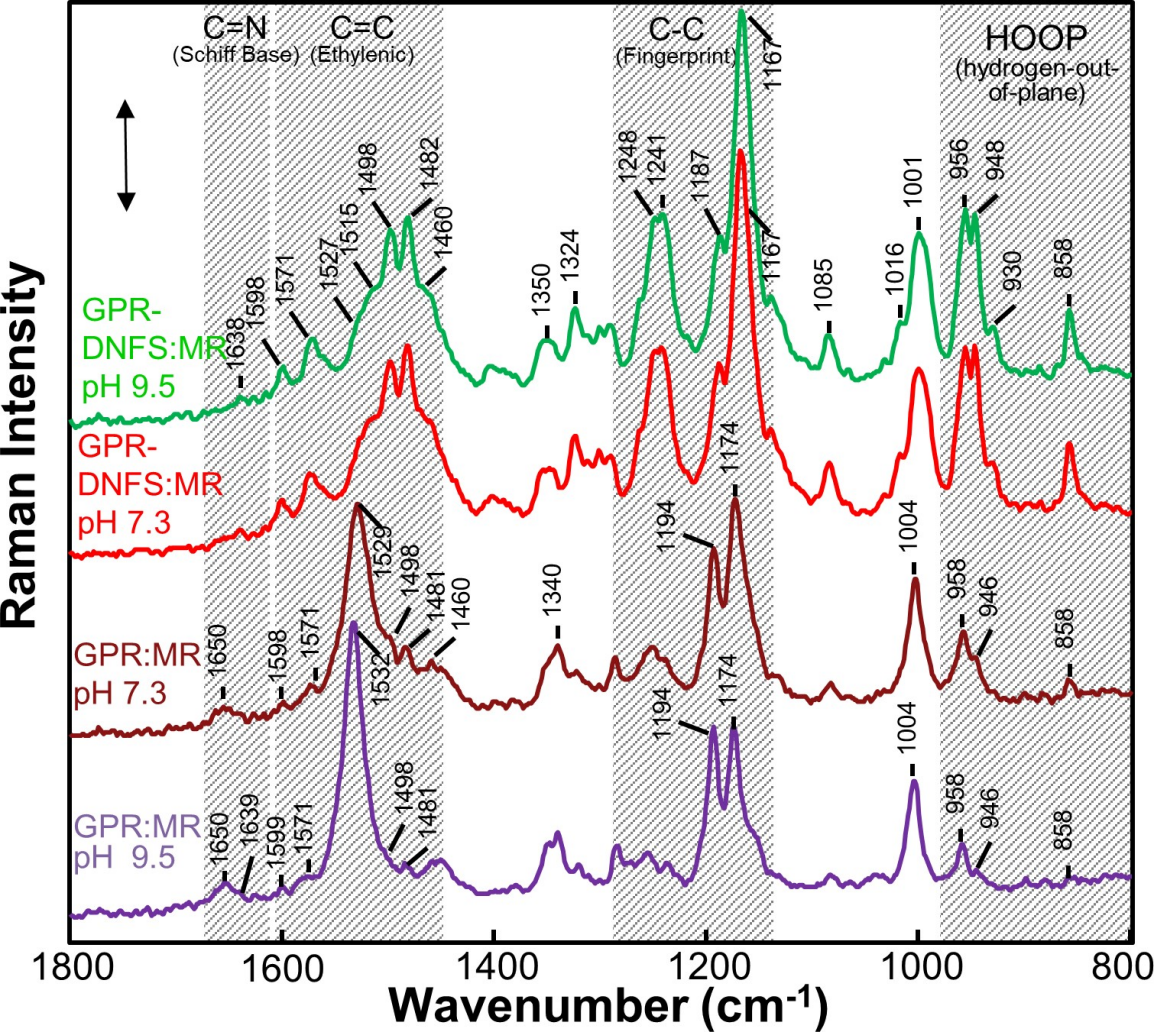
FT-Raman spectra recorded using 1064-nm excitation of GPR:MR and GPR-DNFS: MR at differ pHs in reconstituted membrane vesicles. The GPR:MR spectra at the different pHs were scaled using the 1194 cm^-1^ band and the GPR-DNFS:MR spectra at the different pHs were scaled using the 1167 cm^-1^ band. Laser power and data acquisition times for each sample were GPR-DNFS:MR pH 7.3 (300 mW, 120 min), GPR-DNFS pH 9.5 (300 mw, 113 min); GPR:MR pH 7.3 (400 mW, 113 min); GPR:MR pH 9.5 (400 mW, 226 min). The scale bar shown is for the GPR-DNFS MR pH 7.3 spectrum and corresponds to 0.005 FT-Raman intensity measured by a Ge detector (see Materials and Methods for additional details).

FT-Raman spectra were obtained from suspensions of GPR-DNFS:MR reconstituted membrane vesicles inserted into a capillary (see Materials and Methods). Normally, 785-nm excitation can be used to measure Raman spectra of microbial rhodopsins absorbing in the visible region without strongly exciting photoreactions, thus avoiding photoproducts which can contribute to the spectrum [46, 47]. However, this is not possible for the dominant NIR absorbing components of GPR-DNFS:MR where the 785-nm excitation could result in photoproduct accumulation Furthermore, 785-nm excitation of GPR-DNFS:MR and to a lesser extent 633-nm excitation produced very strong fluorescence emission peaking near 830 nm similar to GPR:MR [49] which strongly interfered with detection of much weaker Raman bands. In contrast, FT-Raman instruments normally utilize 1064-nm excitation which effectively eliminates fluorescence and photointermediate accumulation. In addition, the use of 1064-nm excitation will predominantly resonance enhance the NIR components even though the vibrational modes of rhodopsin chromophores absorbing below 650 nm will still be pre-resonance enhanced [56–58]. This explains why the FT-Raman spectra of GPR-DNFS:MR recorded at pH 9.5 and pH 7.3 are very similar (Fig 4), since the 1064-nm excitation is expected to strongly enhance the NIR absorbing species which are present at both pH 7.3 and 9.5 even though the NIR bands are much weaker at pH 9.5 compared to pH 7.3 (Fig 2).

**Fig 5:**
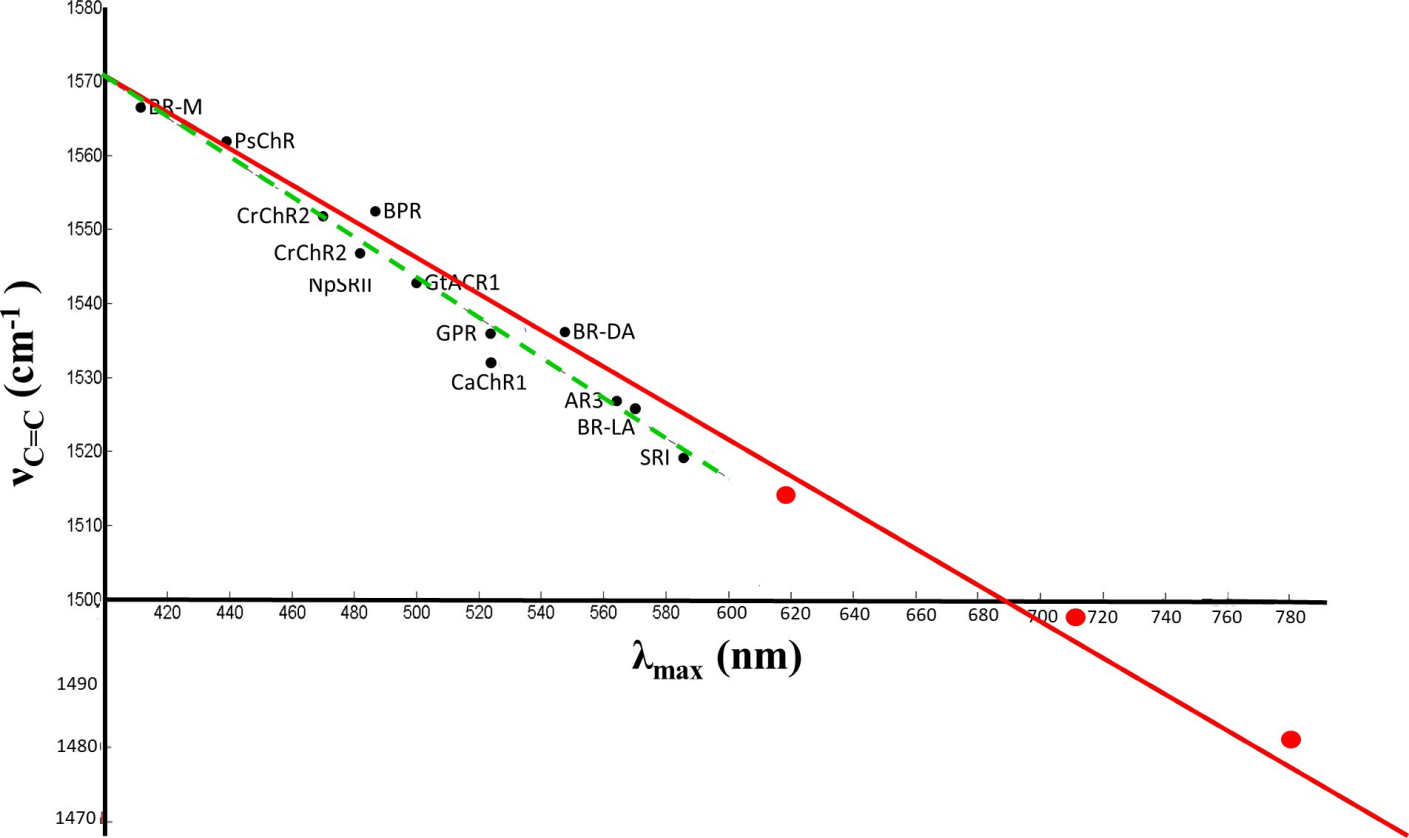
Inverse linear correlation between ethylenic frequency and visible absorption wavelength maximum for several microbial rhodopsins and GPR-DNFS:MR. All wavelength and frequency values for GPR-DNFS:MR (large red dots) are from results reported here (Figs 3 and 4). Additional data points (black dots) include *Ca*ChR1, *Cr*ChR2, *Np*SRII, light-adapted BR [53], light-adapted AR3 [47]; dark-adapted BR [63]; BR M-intermediate [40, 64]; BPR;GPR [46]and SRI [65]. Red solid line shows linear correlation based on visual fit for the GPR-DNFS:MR data. The green dashed line shows the linear correlation previously reported for only visible absorbing microbial rhodopsins. Adapted from Supplementary S3 Fig from [53].

Unlike most other microbial rhodopsins, an intense band(s) is not found in the 1500–1600 cm^-1^ ethylenic C = C stretch region for GPR-DNFS:MR at pH 7.3 and 9.5 (Fig 4). Instead two strong bands appear at 1482 and 1498 cm^-1^ with three weaker bands at 1513, 1529, and 1573 cm^-1^ (see component fit of this region using Fourier self-deconvolution and curve fitting (S3 Fig)). An even weaker band may also be present at 1558 cm^-1^. Interestingly, the 4 most intense bands in the region above 1475 cm^-1^ correlate well with the existence of the 4 major subcomponent absorption bands observed (781, 708, 623 and 563 nm). Based on an extension of the well-known empirical inverse relationship between λ_max_ and ν_C = C_ [50, 53, 59–62], the 1482, 1498, 1513 and 1529 cm^-1^ bands fit well with a linear correlation which includes many other microbial rhodopsins [53] (Fig 5). Thus, these 4 bands can be assigned to the ethylenic vibrations of GPR-DNFS:MR including two that absorb in the NIR.

The C-C stretching region of GPR-DNFS:MR (often referred to as the fingerprint region due to its sensitivity to the retinal configuration) also has an unusual appearance compared to native GPR [46] and other microbial rhodopsins containing the native all-*trans* retinylidene (ATR) PSB chromophore. An extremely intense band appears at 1168 cm^-1^ with a shoulder at 1189 cm^-1^ (Fig 4). The presence of a band near 1198 cm^-1^ is also revealed using curve-fitting procedures (S3 Fig). In contrast, native GPR displays a strong band at 1198 cm^-1^ and weaker band at 1162 cm^-1^ [46] assigned to the localized C_14_-C_15_ and C_10_-C_11_ stretching modes, respectively, in analogy with BR [42, 50, 66].

Interestingly, an intense band near 1169 cm^-1^ is also observed in the RRS of the O intermediate of the light-adapted BR photocycle (sometimes referred to as the O_640_ intermediate because of its λ_max_ near 640 nm) [67]. On the basis of normal mode calculations and isotope labeling, the O_640_ was determined to contains an ATR PSB structure similar to BR_570_ [67]. This intense band is also observed in the time-resolved FTIR difference spectrum of the BR_570_ to O_640_ difference spectrum of the BR mutants Y185F, E204Q and E204D, which all exhibit a slowed O decay [68, 69]. Hence, an intense 1169 cm^-1^ band is considered a “marker band” for an O-like ATR PSB chromophore and reflects a more delocalized electron distribution leading to a red-shifted chromophore absorption.

An additional “marker band” for the O-like state is found in the coupled H-C = C-H hydrogen-out-of-plane (HOOP) mode region. In the RRS of O_640_ three bands appear in this region at 959, 945 cm^-1^ and 977 cm^-1^ [67]. Since a band at 956 cm^-1^ already appears in BR_570_ the 948 and 977 cm^-1^ bands can be considered more characteristic of the O_640_ state. The band near 948 cm^-1^ has also been found to be a unique feature in the FTIR-difference spectrum of the E204Q mutant of BR, which as mentioned above exhibits a slow decaying O-like photointermediate. Strikingly, the two lower frequency bands characteristic of O appear at very similar frequency (957 and 948 cm^-1^) in the case of GPR-DNFS:MR (Fig 4). As discussed later, this again confirms that at least one of the two NIR subcomponent bands is correlated with an O-like state.

In general, the intensity of HOOP modes are highly sensitive to torsion around the single and double bonds in the polyene chain and increase in intensity as the retinal polyene assumes a non-planar configuration, for example as observed in the K intermediate of BR [41, 43, 70], and in the batho-intermediate and some analogs of visual rhodopsin [71–73]. In the case of GPR-DNFS:MR, the split 957 and 948 cm^-1^ band in the FT-Raman spectrum is one of the most intense in the overall spectrum and far more intense than HOOP mode bands seen in GPR:MR (Fig 4). Thus, we conclude that in analogy with the O_640_ intermediate of the BR photocycle, the GPR-DNFS:MR chromophore associated with NIR absorbing components has an all-*trans* PSB conformationally distorted structure (see discussion).

### UV-Vis-NIR absorption and Raman spectroscopy of GPR:MR

**Fig 6:**
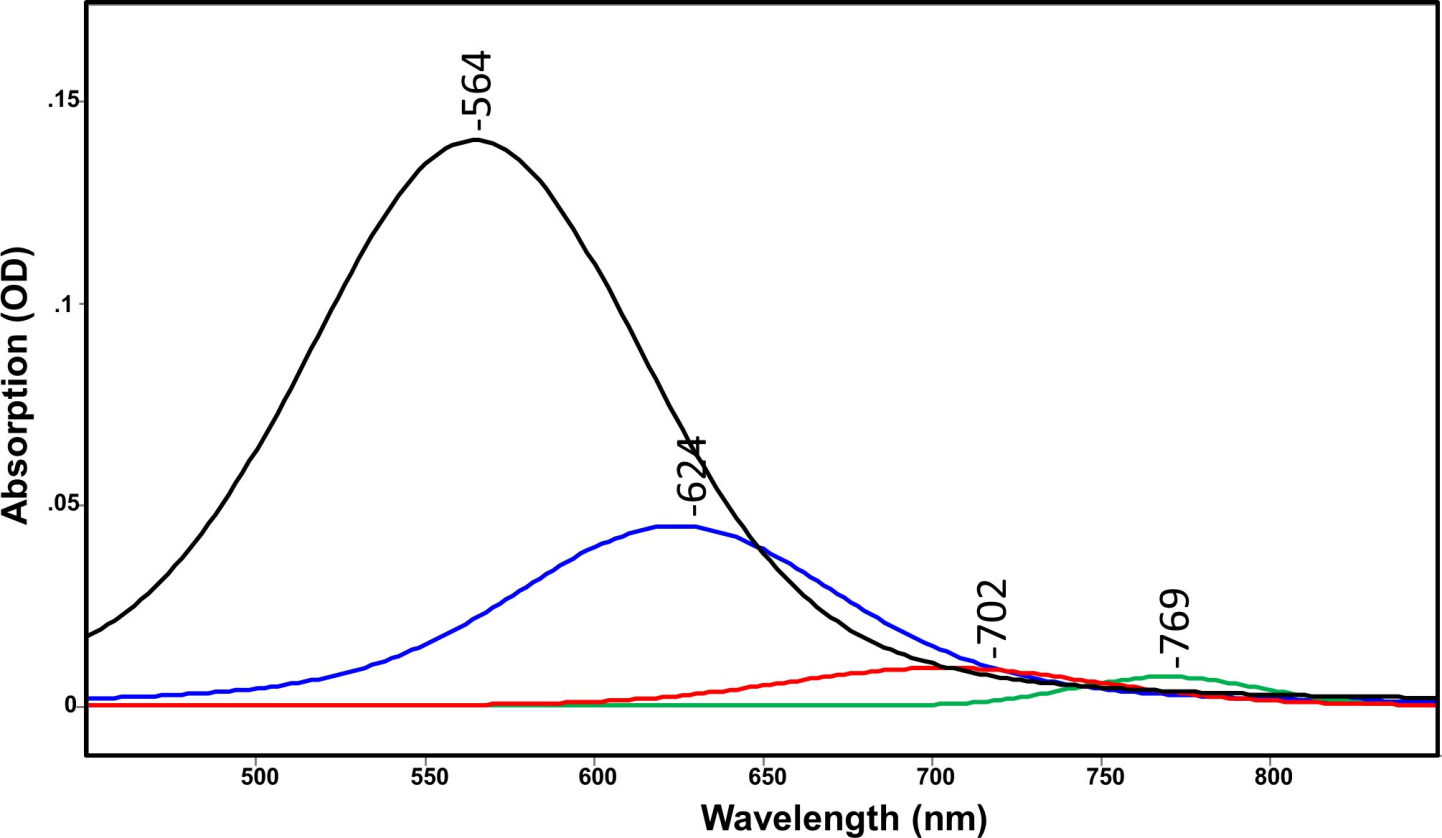
Curve fitted components of absorption spectra shown in Fig 2 for GPR:MR at pH 7. All spectra are scaled to the largest component peak. Curve fitted components are colored black, blue, red and green from lowest to highest wavelength of components peaks. See Materials and Methods for details of curve fitting method.

Compared to GPR-DNFS:MR, the absorption spectrum of GPR:MR (native GPR reconstituted with MMAR as chromophore) exhibits a much smaller red-shift (λ_max_ at 576 and 551 nm at pH 7.3 and 9.5, respectively (Fig 2)). A weak component is also detected at pH 7.3 at 775 nm (Fig 2). Curve fitting of the pH 7.3 spectrum reveals that the major visible component bands are located at 564 and 624 nm with much weaker components in the NIR at 702 and 769 nm (Fig 6). Since all of these bands appear at similar wavelengths as GPR-DNFS:MR, the major effect of the DNFS mutant is to increase the intensity of the NIR components but not significantly shift their wavelengths. In comparison, only visible absorbing bands were detected in GPR:MR at pH 9.5 (Fig 2), in agreement with data reported for alkaline detergent solutions of GPR:MR [35]. Note that due to the higher noise for this measurement, curve fitting was not done.

The FT-Raman spectra of GPR:MR at pH 7.3 and 9.5 exhibit major ethylenic bands at 1529 and 1532 cm^-1^_,_ respectively (Fig 4). The frequency of these bands is consistent with the existence of a predominantly visible absorbing species near 565 nm at pH 7.3 which shifts to a slightly lower wavelength at pH 9.5 based on the empirical correlation between λ_max_ and ν_C = C_ (Fig 5). Small bands also appear in the pH 7.3 and 9.5 spectrum at 1498 and 1482 cm^-1^, almost the identical frequency as the ethylenic bands that appear with much higher intensity in GPR-DNFS:MR spectra (Fig 4). Additional bands associated with the NIR absorbing species are also detected at 1174 and 948 cm^-1^ assigned to the C-C stretching and HOOP modes, respectively. However, all of these bands are weaker in the pH 9.5 spectra compared to pH 7.3 (see below). This again confirms the existence of a small amount of the NIR absorbing species in GPR:MR which is similar to the NIR species in GPR-DNFS:MR. Note that even though these NIR absorbing species are not detected in the visible absorption at pH 9.5, their presence is significantly enhanced in the Raman spectrum relative to the visible absorbing species due to the proximity of the 1064-nm excitation.

An interactive difference spectrum between the FT-Raman spectra of GPR:MR at pH 7.3 and pH 9.5 (e.g. pH 7.3 –pH 9.5) (S4 Fig) has several features which are remarkably similar to light-adapted BR to O_640_ time-resolved FTIR difference spectrum of Y185 (S4 Fig) [68]. For example, both spectra exhibit a strong positive band near 1169 cm^-1^ in the fingerprint region and near 951 cm^-1^ in the HOOP mode regions. Note however, that the positive bands in the ethylenic region are different, reflecting the conversion to a 640 nm absorbing O-like species in Y185F (1509 cm^-1^) (upper trace) and NIR absorbing species of GPR:MR (1495 and 1484 cm^-1^). Note that for the pH difference spectrum of GPR:MR an additional band appears at 858 cm^-1^ in the HOOP mode regions which also appears in the FT-Raman spectra of GPR-DNFS:MR and GPR:MR (Fig 4). This band may represent an isolated = C-H HOOP vibration distinctive for the NIR absorbing species.

### UV-Vis-NIR absorption and Raman spectroscopy of GPR-D97N:MR

**Fig 7:**
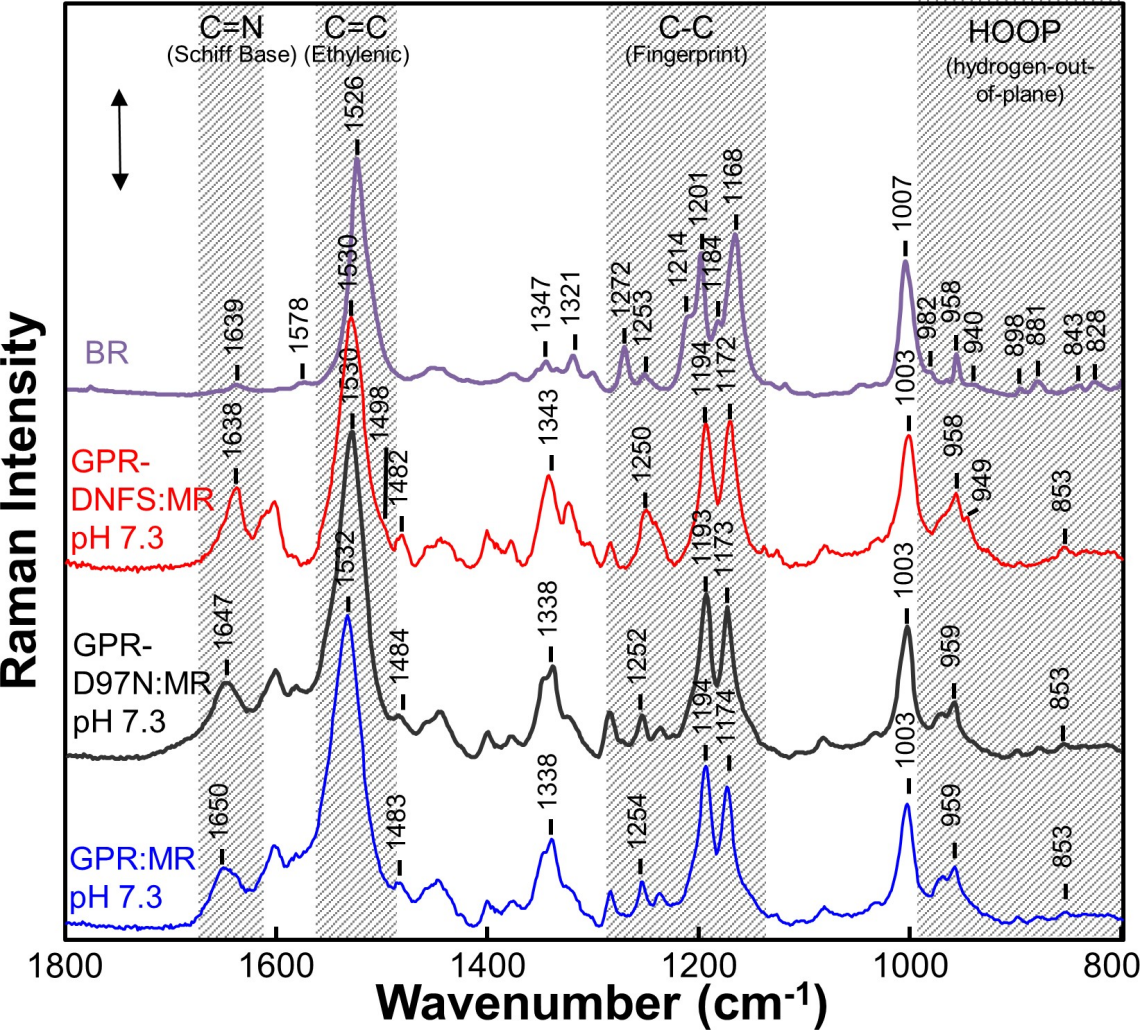
Comparison of the RRS of BR with native A1 retinal chromophore and various GPR with MMAR chromophore. Data was recorded at room temperature using 532-nm laser excitation for GPR:MR, GPR-D97N:MR and GPR-DNFS:MR using 2.8 mW laser power. The BR spectrum, reported previously [75], was recorded using 785-nm excitation. Spectra were not smoothed and were scaled approximately using the intensity of the peaks in the fingerprint region. A background spectrum of the borosilicate capillary and buffer was subtracted from the sample. The BR spectrum is reproduced from ref. [75]. The scale bar shown is for the GPR-DNFS:MR pH 7.3 spectrum and corresponds to 500 counts of Raman scattering intensity. Additional details are given in Materials and Methods.

In order to assess the effects of the D97N mutation on the MMAR chromophore, we measured the UV-Vis-NIR absorption of GPR-D97N:MR along with its RRS using 532-nm excitation (see Materials and Methods). The visible absorption maximum at pH 7.3 of D97N with the native retinal chromophore (A1) peaks near 550 nm [28, 50, 74] and is shifted to 632 nm for GPR-D97N:MR with a very small NIR band appearing at 790 nm (Fig 2). RRS measured using 532-nm laser excitation is expected to resonance enhance components which absorb mainly in the visible, especially those species with λ_max_ near 532 nm. For example, the 532-nm excited RRS of GPR-DNFS:MR at pH 7.3 exhibits a strong ethylenic band at 1530 cm^-1^ (Fig 7) which corresponds to the weak subcomponent absorption band at 559 nm (Fig 3B). On the other hand, the major absorption bands at 708 and 778 nm are only weakly resonance enhanced and give rise to the two small bands at 1498 and 1482 cm^-1^ which are more strongly enhanced in the 106- nm excited FT-Raman spectrum (Fig 4).

The 532 nm excited RRS spectrum of GPR-D97N:MR (Fig 7) is very similar to that of GPR-DNFS:MR and of GPR:MR including similar bands in the ethylenic, fingerprint and HOOP mode regions indicating that the sub-species absorbing near 560 nm is present even though the absorbance band peaks at 632 nm (Fig 2). Interestingly, a small subcomponent band in the RRS is found at 1484 cm^-1^ which corresponds approximately to the 790 nm absorption bands (Fig 2). This confirms that GPR-D97N:MR does produce at least one NIR absorbing component, although much weaker than in GPR-DNFS:MR.

## Discussion

The ability to shift the visible absorbance of microbial rhodopsins into the NIR has many advantages for *in vivo* optogenetic applications. The scattering of light by biological tissues containing fibroid structures, cellular membranes, lipid globules and protein complexes increases non-linearly at lower wavelength and thus limits light penetration. In addition, visible light is strongly absorbed by endogenous molecules such as hemoglobin, cytochromes, melanin and quinone derivatives. In contrast, relatively little absorption and much less scattering occurs in the so-called tissue transparency window from ~700–900 nm [76]. Thus, effective optogenetic monitoring and control of neurons and other cells in tissues is severely limited to depths of only a few mm for most *in vivo* applications without using NIR wavelengths and advanced NIR optical techniques which can take advantage of this transparency window [76–79]. In this regard, the development of NIR excitable and emitting microbial rhodopsin voltage sensors and membrane voltage modulators would be particularly attractive for monitoring or manipulating electrical activity in the brain, especially to simultaneously measure the activity of millions of individual neurons over large volumes of tissue.

A variety of genetically encoded voltage indicators (GEVIs) have been developed including those based on fusion of fluorescent proteins to transmembrane voltage sensing domains and on native and mutant microbial rhodopsin proton pumps [80]. For example, the first microbial rhodopsin GEVI developed is based on the GPR D97N mutant (referred to as a Proteorhodopsin Optical Proton Sensor or PROPS) which when excited by a 632-nm HeNe laser fluoresces around 735 nm [30, 74, 80]. Similar to the homologous D85N BR mutant, the substitution of an Asn for an Asp at position 97 neutralizes the retinal PSB counterion causing a red-shift in the λ_max_ from 525 nm to 555 nm (S2 Fig) and also blocks proton transport [81]. A similar neutralization of the SB counterion occurs in light-adapted BR at low pH (~3) to produce the red-shifted acid-blue membrane [82–84].

Since GPR and variants including PROPS do not express well in mammalian cells, a variety of improved microbial rhodopsin GEVIs have been developed [31–33, 80]. One example is the series of QuasARs (e.g. QuasAR1,2) which are evolved from the archaerhodopsin 3 (AR3) proton pump with several mutations including a neutralized Schiff base counterion [31]. Recently, an improved GEVI referred to as Archon1 was developed using robotic multidimensional directed evolution approach which exhibits large and linear fluorescence changes in response to voltage fluctuations [33]. However, all of these GEVIs are still excited using visible light.

This study focuses on a recently developed NIR absorbing microbial rhodopsins based on a mutant of GPR (D212N/F234S) regenerated with the retinal analog 3-methylamino-16-nor-1,2,3,4-didehydroretinal (MMAR) [35] (S1 Fig). Similar to the A2 retinal analog 3,4-dehydroretinal, the modified retinal promotes charge delocalization, normally largely limited to the polyene chain, by extending the conjugated system into the modified ring (S1 Fig). In addition, the ring substituted polar methylamino group of MMAR provides an additional site for the protein to modulate the absorption wavelength. As demonstrated [35, 85] (Fig 2), at a pH <8 GPR-DNFS:MR absorbs strongly in the NIR region between 700 and 800 nm and emits strongly near 830 nm similar to GPR:MR [49]. This raises the possibility that GPR-DNFS:MR can be used as an NIR GEVI. Recently, a second NIR absorbing microbial rhodopsin was developed by regenerating an evolved AR3 mutant with a merocyanine retinal analog [37]. However, in both cases the molecular basis of the extreme opsin shifts is not understood and likewise the principles to bioengineer further optimized NIR rhodopsins for optogenetic applications are unknown.

### Differences between the Properties of MR pigments in bilayer membranes and DDM micelles

Earlier absorption measurements of GPR:MR and variants such as GPR-DNFS:MR and GPR-D97N:MR were performed in DDM detergent micelles [35, 85]. In general, such micellar solutions displayed NIR absorption bands which predominated at pH below 7. However, much larger amounts of the NIR components were observed at higher pH [35, 85]. One possible reason for this difference is that the current measurements were performed with these proteins reconstituted into ECPL lipid bilayer membranes which is closer to the native GPR lipid bilayer environment in the plasma membrane. In general, differences in the environment of microbial rhodopsins, especially between detergent micelles and lipid bilayer membrane, can result in significant alterations in their properties including absorption spectrum, oligomerization state, response to pH, ability to regenerate a functional protein, and photocycle kinetics. One well-studied example is light-adapted BR whose absorption blue shifts from 570 nm in the native purple membrane form which consists of trimeric oligomers to around 550 nm when solubilized as a monomer in detergent or even bilayer form [86]. In the case of proteorhodopsin, oligomerization and cooperative interactions between monomers have been observed depending on the environment [23, 55].

An additional factor is that our absorption measurements were performed mainly on ECPL bilayer membrane vesicles incorporated into hydrated multilamellar films. Higher salt concentration is expected in such films due to the partial drying of the buffer during the film formation. This higher salt concentration could suppress the NIR components by enabling an anion to enter the active site, thus producing the equivalent of BR acid-purple membrane [82] (see discussion below). Alternatively, dichroism effects, which can occur in oriented multilamellar films can lead to suppression of the NIR component of the chromophore if its MMAR dipole moment had larger out-of-plane components compared to the visible chromophore [87].

It should also be noted that the curve fitting procedure (see Materials and Methods) used to analyze the different levels of visible/NIR band contributions is likely to be only approximate. One reason is that cyanine-like NIR absorbance bands need not be symmetrical [88, 89]. Furthermore, retinal and retinal protein absorbance bands have lower intensity blue-shifted β-peak extensions of the main band. As a consequence, the β -peak may account for the presence of multiple peaks in the visible and NIR region and their potential overlap with α-bands could lead to errors in the determination of the wavelength and level of putative individual species.

### Similarities between the NIR absorbing form of GPR-DNFS:MR and the BR O_640_ photointermediate

This study reveals several similarities between the acidic form of GPR:MR and GPR-DNFS:MR and O_640_ which constitutes the last photointermediate in the BR photocycle [1, 2, 90]. First, both have red-shifted absorptions compared to their “parent form”; BR_570_ in the case of O_640_ and the alkaline form in the case of GPR:MR and GPR-DNFS:MR. Second, in both cases, the red-shift most likely requires or is strongly enhanced upon neutralization of the Schiff base counterion (D85 and D97, respectively) due to its protonation. Third, GPR-DNFS:MR exhibits vibrational bands previously identified as characteristic of the O_640_ photointermediate. This includes an intense band at 1169 cm^-1^ in the fingerprint region, and a second intense band near 948 cm^-1^ in the coupled-HOOP mode region. These bands indicate that like the O_640_ photointermediate, the NIR GPR-DNFS:MR chromophore adopts an *all-trans* PSB configuration with a distorted non-planar polyene chain due to torsions in the conjugated double bond system. Strong torsional distortion is also observed for the K photointermediates of BR_570_ and other microbial rhodopsins, but in this case the retinal structure has a 13-*cis* PSB configuration [3], and similarly for the 11-*cis* ground structure and all-*trans* batho-intermediate of visual rhodopsin [71–73].

The existence of a stable O-like state before the photocycle is initiated is not unprecedented. For example, the BR mutant Y185F was found, based on static and time-resolved UV-Vis absorption spectroscopy to exist in a pH dependent equilibrium between a “purple” species similar to BR_570_ and a blue O-like species [91, 92]. A low-temperature FTIR-difference study of Y185F subsequently revealed that the chromophore vibrational bands characteristic of O_640_ are also common to the blue state of Y185F. Furthermore, like O_640_ this blue Y185F state has a PSB and lacks an M intermediate in its photocycle [93].

Additional examples of red-shifted O-like species have been found for the BR mutants D85T and D85S. D85T exhibits a red-shifted stable blue form similar to acid-blue due to neutralization of the SB counterion [94, 95]. X-ray crystallography of D85S reveals features which support its similarity to O_640_ photointermediate [96, 97]. Interestingly, unlike the M_2_ and N photointermediates of BR, where structural changes occur mainly on the extracellular side of the membrane and the chromophore is in a 13-*cis* configuration, during O_640_ formation structural changes occur on the cytoplasmic side of the membrane and are most likely associated with uptake of a proton from a water molecule to reprotonate Asp96, the proton donor to the SB [68]. Several mutants also exhibit a slowed O decay such as E204Q which is attributed to the inhibition of deprotonation of Asp85 during the last step (O_640_ → BR_570_) of the photocycle [98]. Again a characteristic intense C-C stretch mode (1169 cm^-1^) and HOOP mode (945 cm^-1^) appears indicative of a non-planar polyene chain associated with O_640_ [69].

### Similarity between the 560-nm absorbing component of GPR-DNFS:MR and BR_570_

Our results indicate that the component of GPR-DNFS:MR as well as GPR and GPR-D97N absorbing near 560 nm has an all-*trans* PSB structure at both high and low pH which is similar to the ground state of light-adapted BR (BR_570_) [50]. In general, RRS of microbial rhodopsins using 532-nm excitation is expected to enhance most strongly the vibrations of species absorbing nearest this wavelength. In the case of GPR-DNFS:MR this is the ~560 nm component which is favored at pH 9.5 but is still detected at pH 7.3 and 5 (Fig 3A–3C). In agreement. at pH 7.3 a strong ethylenic band appears at 1530 cm^-1^ which corresponds to a species absorbing near 560 nm (based on the λ_max_ vs. ν_C = C_ empirical correlation, Fig 5). The strong similarity between the RRS of this species and BR_570_ (Fig 7) indicates that both have a very similar structure, i.e. an *all-trans* PSB chromophore with a negatively charged SB counterion which is Asp97 in the case of GPR-DNFS:MR.

A second possibility is that the 560-nm component of GPR-DNFS:MR is similar to the acid-purple form of BR. This form of BR is produced from acid-blue by lowering the pH below 2 so that a hydroxide enters the active site or by increasing the salt concentration so that an anion (e.g. chloride or bromide) enters the active site to serve as the SB counterion [82]. Since the RRS of acid-purple is almost identical to BR_570_ [67], it is difficult to distinguish the two forms on the basis of the RRS or visible absorption. In the case of GPR-D97N:MR, where the Asp 97 counterion is substituted with a neutral Asn residue, it is possible that the acid-purple form accounts for the BR-like RRS. Interestingly, in the case of D85T, the acid-purple form functions as a light driven anion pump similar to halorhodopsins [94, 95].

When retinal or an analog retinal such as MMAR is incorporated into a microbial rhodopsin via a positively charged PSB (iminium) linkage, a variety of factors can influence the absorption [12, 99]. These include: i) the detailed interactions of the protein with the PSB which can involve the proximity of one or more PSB counterions (e.g. Asp97 in the case of GPR); ii) the interaction of water molecules located near the PSB and counterion(s), such as in the case W402 for BR_570_ [100]; iii) the polarity of residues lining the retinal pocket which affect the change in dipole moment between the ground and excited state; iv) the detailed conformation of the retinal analog including twists around single and/or double bonds which can cause the chromophore to assume a non-planar structure [101]; and v) polarity or voltage gradient over the entire protein.

In addition to these factors, analog retinals such as A2 (3,4-dehydroretinal) and MMAR allow increased delocalization of π -electrons over the polyene chain resulting in a red-shift of the visible absorption [35] (S1 Fig). In the case of MMAR, the presence of a second nitrogen can effectively enhance delocalization of the positive charge, which is normally localized near the SB with the lysine-nitrogen owing to a strong interaction with a negatively charged counterion complex. Upon protonation of the main counterion (Asp97), this charge can be more effectively delocalized towards the methylamino group, thus generating an unprecedented large red-shift in the absorbance band [85].

## Conclusions

Based on the results presented here, a more detailed picture emerges of the molecular structure of one of the first microbial rhodopsins variants discovered which exhibits its strongest absorption in the NIR. While the combination of the double mutant D212N/F234S and analog chromophore MMAR (GPR-DNFS:MR) produce a pronounced redshift to the NIR, almost identical NIR absorbing forms were detected, but at significantly reduced intensity, when MMAR is substitute for the native A1 retinal chromophore in wild-type GPR (GPR:MR) and the voltage sensing mutant D97N (D97N;MR).

In the case of GPR-DNFS:MR, the simplest explanation for the extreme red-shift is that these factors (the MR chromophore substitution and DNFS mutation) synergistically enhance the charge delocalization in the MMAR chromophore. One key feature, as indicated by the FT-Raman data, is a non-planar geometry of the chromophore, due to twists in the conjugated polyene segment, similar to the structure of the native A1 retinal chromophore previously established for the O_**640**_ photointermediate of BR. In analogy with the O_640_ photointermediate, GPR-DNF:MR is predicted to have an all-*trans* chromophore with a protonated PSB counterion.

**Fig 8:**
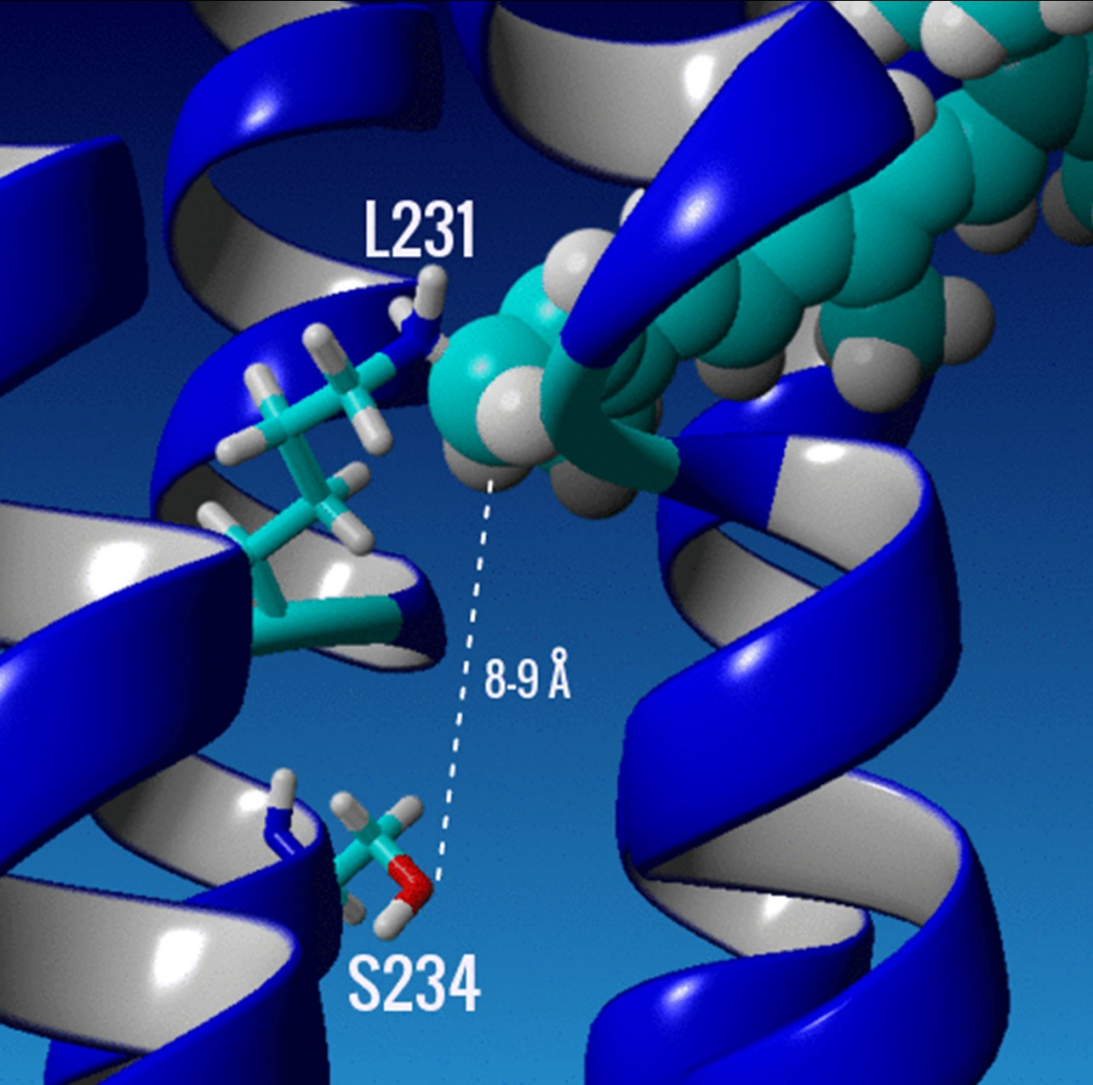
Close up of the retinal binding pocket in a homology model of GPR-DNFS. The K231 and the mutant S234 on helix G (central blue helix in picture) are selectively displayed as stick models of the amino acid side chains, while the A1 retinal chromophore is displayed as a space filled residue in cyan. Dashed line shows estimated distance between the hydroxyl oxygen of S234 and the C15 carbon of the SB of the A1 retinylidene chromophore. The homology model was generated using sensory rhodopsin II (SRII) as a template (PDB 1H2S) [102] with the program YASARA (www.yasara.org) as described previously in [35] and chapter 2 of ref. [85].

A direct effect of the F234→S234 substitution on the chromophore, especially near the PSB is unlikely since the distance of S234 on helix G from the nitrogen in the PSB is approximately 9 Å based on a homology model (Fig 8). Indirect effects could include the disruption of a network of water molecules located near the SB or a change in the relative position of helix G which could cause a movement of the MMAR chromophore relative to that in wild type GPR. Additional studies involving a variety of biophysical approaches, including FTIR-difference spectroscopy and x-ray crystallography, will be necessary to further elucidate the molecular basis of the extreme red-shift exhibited in GPR-DNFS:MR as well as other microbial rhodopsin variants that absorb in the NIR.

## Supporting information

S1 Fig Chemical structure of native and analog retinals. (a) the native A1 retinal found in microbial rhodopsins; (b) analog retinal A2 (3,4-dehydroretinal); and (c) MMAR retinal (3-methylamino-16-nor-1,2,3,4-didehydroretinal) (adapted from Fig 1 of ref. [35]).
(TIF)

S2 Fig Absorption spectra from 250–950 nm of GPR:A1, GPR-D97N:A1 and GPR-D97N:MR at pH 7.3. All GPRs were reconstituted into *E*. *coli* polar lipids membrane vesicles and used to produce fully hydrated multilamellar films deposited on BaF_2_. All spectra were scaled using the 281 nm absorption band. DNFS is abbreviation for the mutant D212N/F234S and MR for MMAR chromophore. Absorption (OD) scale shown is for GPR-D97N:A1 where division on Y-axis are 0.2 OD.
(TIF)

S3 Fig **Curve fit of the component bands contributing to the FT-Raman spectrum of GPR-DNFS:MR at pH 7.3 in (A) 1400–1650 cm**^**-1**^ **region and (B) 1100–1280 cm**^**-1**^ **region.** Raman intensity scale shown is for the unfitted GPR-DNFS:MR spectrum (red). Fitted spectrum is shown in black. Unfitted spectrum over full spectral range shown in Fig 4.
(TIF)

S4 Fig Comparison of GPR:MR FT-Raman pH difference spectrum and globally fitted time-resolved BR → O difference spectrum of the BR mutant Y185F. (A) Spectrum calculated by interactively subtraction of the pH 9.5 GPR:MR FT-Raman spectrum from the pH 7.3 GPR:MR spectrum (both spectra shown in Fig 4) (pH 7 spectrum–pH 9.5 spectrum). (B) O-BR time-resolved difference spectrum for mutant Y185F (O_640_ Y185F spectrum–BR_570_ Y185F spectrum) (see [68]). Scale shown is Raman intensity calculated for the FT-Raman difference spectrum.
(TIF)
