# Supplementary figures and images for: Raman spectroscopy of a near infrared absorbing proteorhodopsin: Similarities to the bacteriorhodopsin O photointermediate
Source: PLoS One. 2018 Dec 26;13(12):e0209506. doi: 10.1371/journal.pone.0209506 (PMC6306260; doi:10.1371/journal.pone.0209506)

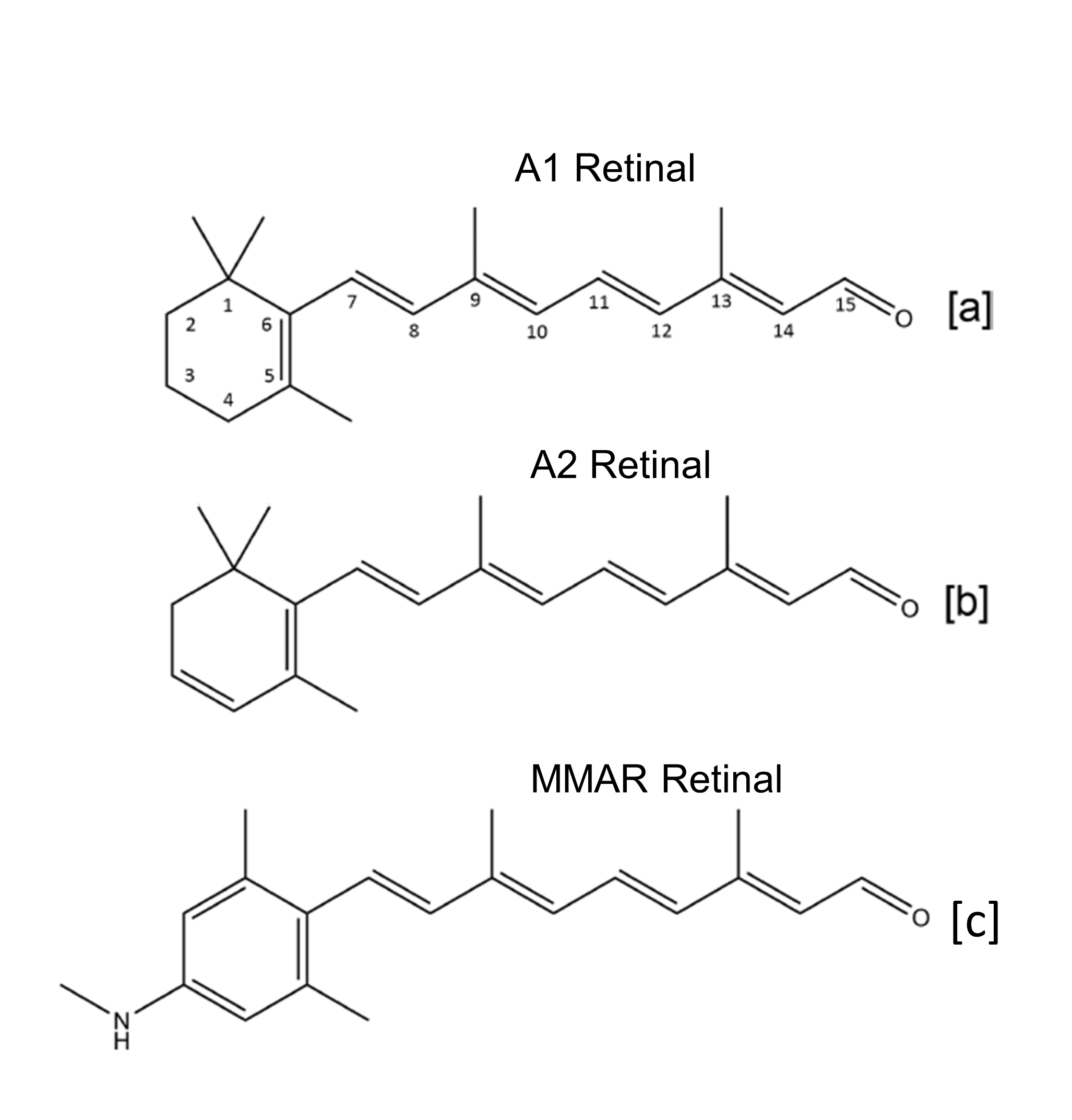

Supplement: S1 Fig — (a) the native A1 retinal found in microbial rhodopsins; (b) analog retinal A2 (3,4-dehydroretinal); and (c) MMAR retinal (3-methylamino-16-nor-1,2,3,4-didehydroretinal) (adapted from Fig 1 of ref. [35]). (TIF) [file pone.0209506.s001.tif]

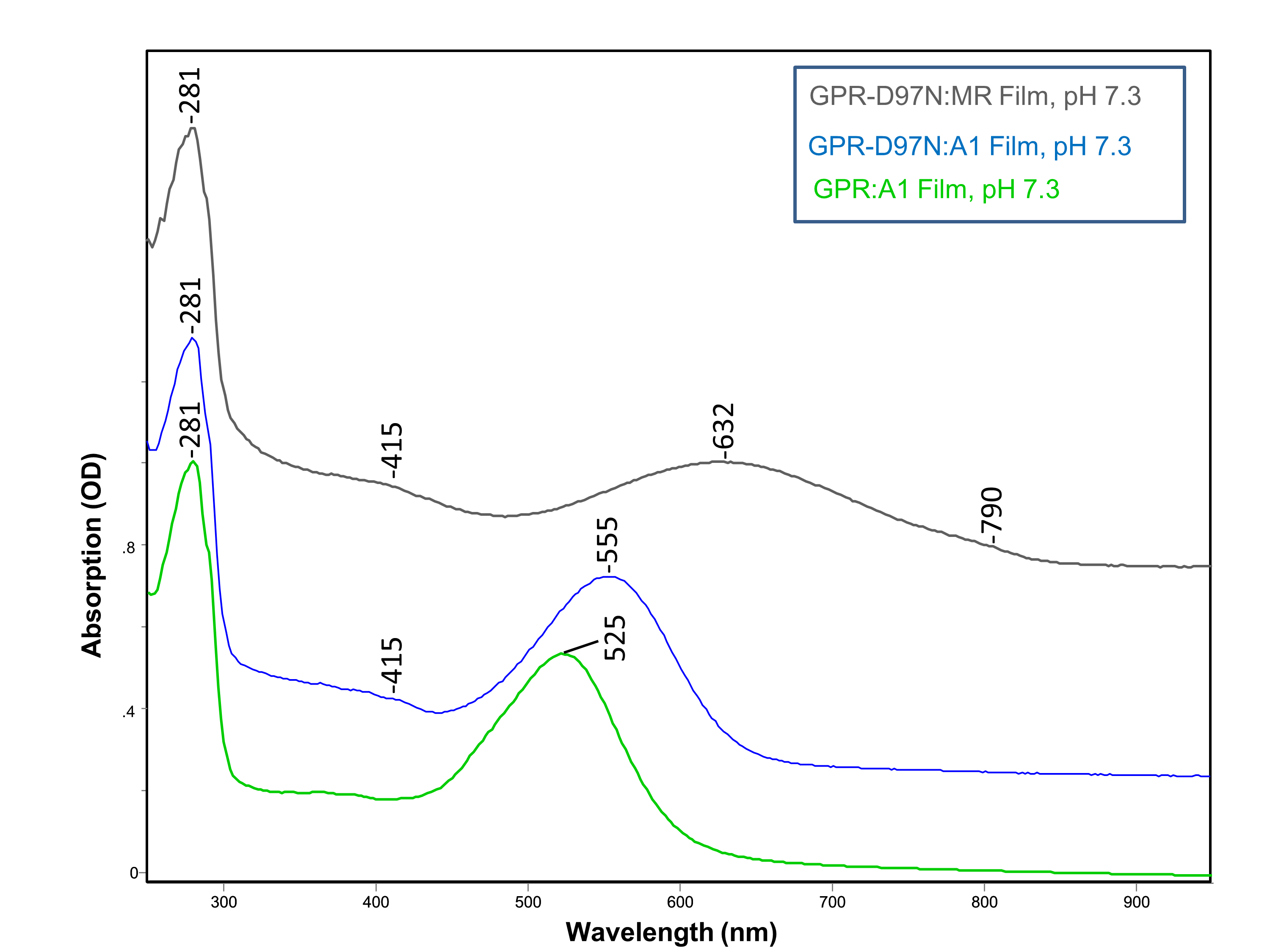

Supplement: S2 Fig — All GPRs were reconstituted into E. coli polar lipids membrane vesicles and used to produce fully hydrated multilamellar films deposited on BaF2. All spectra were scaled using the 281 nm absorption band. DNFS is abbreviation for the mutant D212N/F234S and MR for MMAR chromophore. Absorption (OD) scale shown is for GPR-D97N:A1 where division on Y-axis are 0.2 OD. (TIF) [file pone.0209506.s002.tif]

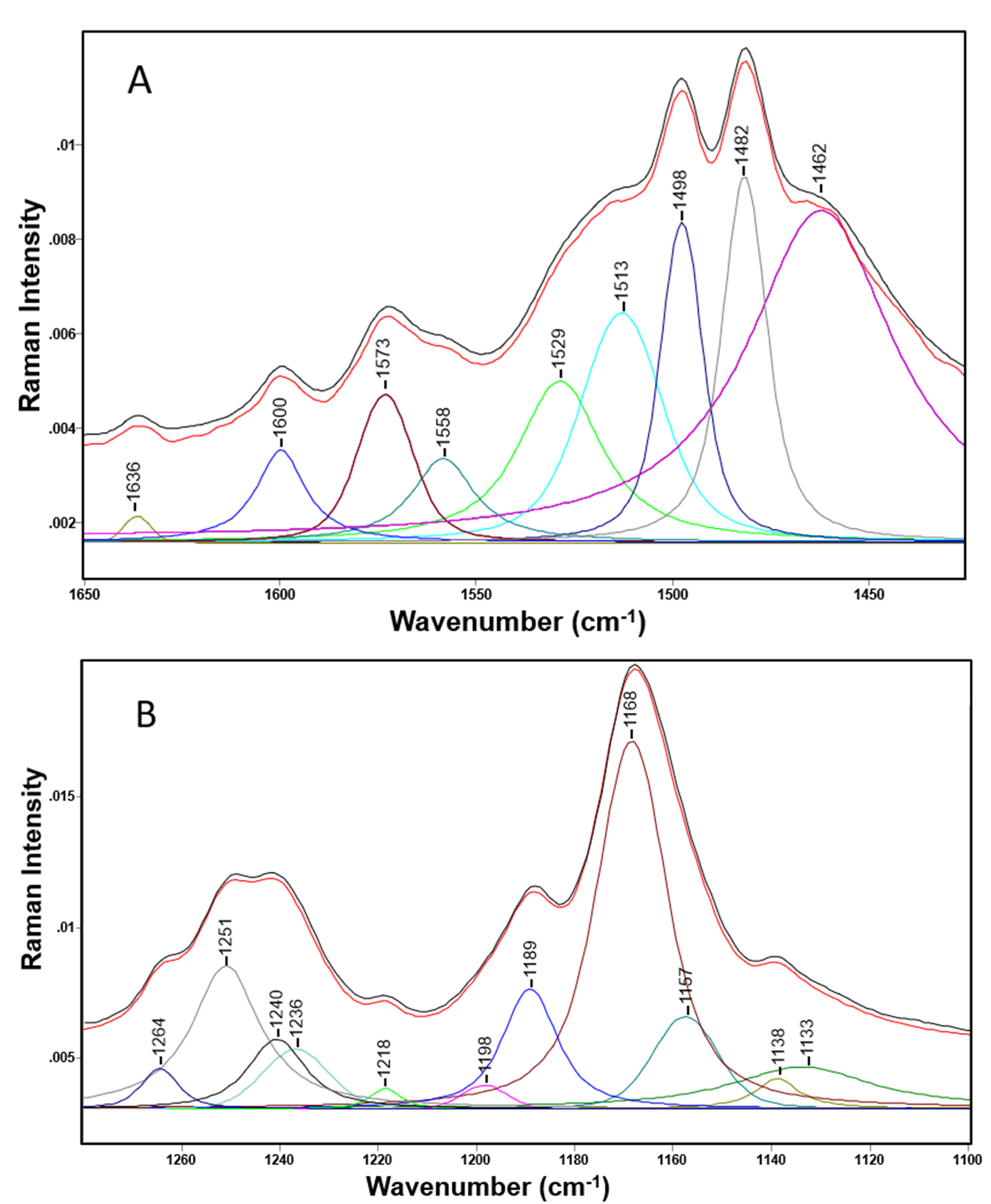

Supplement: S3 Fig — Curve fit of the component bands contributing to the FT-Raman spectrum of GPR-DNFS:MR at pH 7.3 in (A) 1400–1650 cm-1 region and (B) 1100–1280 cm-1 region. Raman intensity scale shown is for the unfitted GPR-DNFS:MR spectrum (red). Fitted spectrum is shown in black. Unfitted spectrum over full spectral range shown in Fig 4. (TIF) [file pone.0209506.s003.tif]

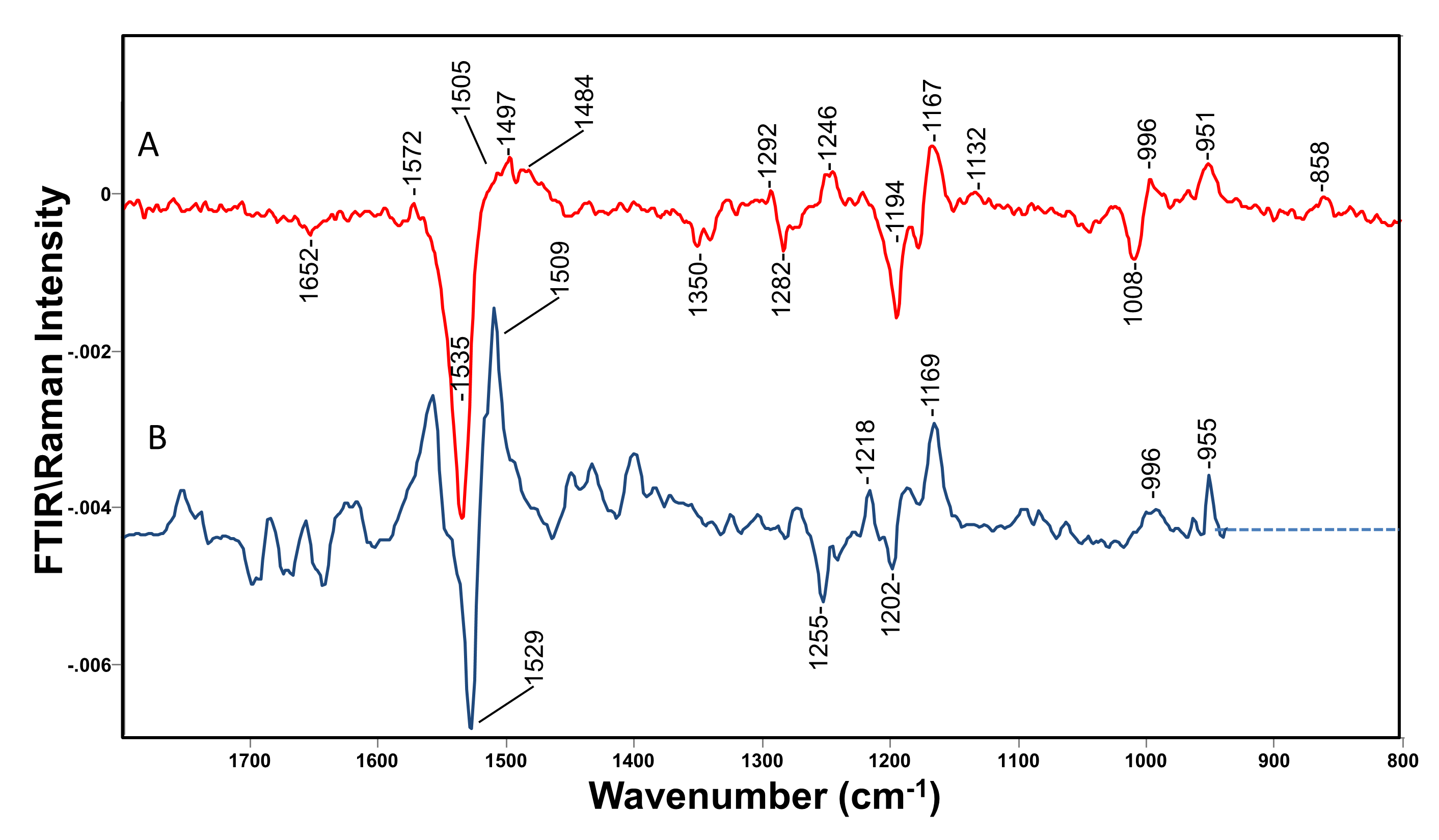

Supplement: S4 Fig — (A) Spectrum calculated by interactively subtraction of the pH 9.5 GPR:MR FT-Raman spectrum from the pH 7.3 GPR:MR spectrum (both spectra shown in Fig 4) (pH 7 spectrum–pH 9.5 spectrum). (B) O-BR time-resolved difference spectrum for mutant Y185F (O640 Y185F spectrum–BR570 Y185F spectrum) (see [68]). Scale shown is Raman intensity calculated for the FT-Raman difference spectrum. (TIF) [file pone.0209506.s004.tif]
